# Training emergency physicians in sex- and gender-based medicine: assessing attitudes of program directors and residency graduates

**DOI:** 10.1186/s13293-016-0098-2

**Published:** 2016-10-14

**Authors:** Tracy E. Madsen, Alyson J. McGregor

**Affiliations:** Department of Emergency Medicine, Alpert Medical School of Brown University, 55 Claverick Street, 2nd floor, Providence, RI 02908 USA

**Keywords:** Gap analysis/needs assessment, Gender, Emergency medicine, Curriculum

## Abstract

**Background:**

Sex and gender influence disease presentation, treatment, healthcare access, and long-term outcomes. It is uncertain to what extent sex- and gender-based medicine (SGBM) content has been integrated into emergency medicine (EM) residency curricula. We aimed to determine if SGBM is being taught in EM residency training, if EM residency program directors (PDs) declare SGBM a curriculum priority, and if recent graduates (RGs) of EM residency programs declare SGBM as relevant to their practice.

**Methods:**

Two hundred twenty-six RGs and 54 PDs of US ACGME EM residency programs completed a web-based survey. Descriptive statistics were used to describe RGs’ attitudes towards whether they had received instruction in SGBM overall and in specific content areas and attitudes about the relevance of SGBM to EM practice. Descriptive statistics were also used to describe whether SGBM was considered a curriculum priority by PDs and potential barriers to implementing SGBM into curricula.

**Results:**

43.2 % of RGs felt they received adequate training on gender differences in emergent conditions. Only 16.3 % of PDs believed gender differences in disease presentation were a curriculum priority. In contrast, the majority (59.5 %) of RGs felt that gender differences in emergency conditions were relevant to their practice. PDs listed completing curricular demands (76.6 %), lack of evidence-based content (53.2 %), and lack of faculty interest (36.2 %) as the largest obstacles to curriculum integration.

**Conclusions:**

Over half of the RGs of ACGME EM residencies felt that their instruction in SGBM was not adequate, and SGBM was not reported as a consistent priority among PDs.

**Electronic supplementary material:**

The online version of this article (doi:10.1186/s13293-016-0098-2) contains supplementary material, which is available to authorized users.

## Background

Sex and gender affect many facets of acute disease including pathophysiology, risk factors, clinical presentation, treatment, and outcomes. For example, sex and gender differences exist in disease conditions including acute coronary syndrome, stroke, COPD, and substance abuse, conditions that account for large numbers of emergency department visits each year [1–4].

The field of women’s health has classically focused on reproductive health and has not consistently included content on sex- and gender-based medicine (SGBM) despite an increasing amount of evidence that clinically important sex- and gender differences exist in a wide variety of acute conditions. Though not systematically, there have been small-scale efforts to integrate SGBM into the curricula for Ob-Gyn and Internal Medicine residents [5–8]. One internal medicine residency program described the development and implementation of a multidisciplinary women’s health program with a small component of content on gender differences and disparities [5]. Furthermore, curricula integrated into internal medicine training programs have focused on conditions specific to women including breast cancer and gynecological conditions, with a paucity of content on sex and gender differences in conditions that affect both genders [5, 7]. With the exception of a recent publication on the development of SGBM curriculum for residents and fellows [9], the extent to which SGBM and women’s health content has been integrated into emergency medicine (EM) residency curriculum is unknown.

The objectives of our study were (1) to determine if sex and gender differences in diagnosis and treatment of common emergency complaints are being taught in EM residency training, (2) to determine if EM residency program directors (PDs) declared this a curriculum priority, and (3) to determine if recent graduates (RGs) of EM residency programs declared sex- and gender-based curriculum as being relevant to their practice.

## Methods

### Study population/recruitment

We performed cross-sectional, web-based surveys of PDs and RGs of United States (US) Accreditation Council for Graduate Medical Education (ACGME) EM residency programs. For PDs, in January 2008, an invitation email with a link to the online survey was sent to the 140 PDs of US ACGME-approved EM residency programs listed on the Society of Academic Emergency Medicine’s (SAEM) 2007 ACGME list of approved residency programs. This was followed by two email reminders 2 and 4 weeks after the initial email invitation. For RGs, a postcard with a link to the online survey (specific to RGs) was mailed to the 2023 US EM residency graduates on the American College of Emergency Medicine’s (ACEP) young physician’s mailing list, a list of US residency graduates in their first two post-graduate years. The initial postcard mailing was followed by two subsequent reminder postcards between February and March 2008. Both PDs and RG respondents were entered into a raffle for $100 Amazon.com gift cards. The study was approved by the institutional IRB.

### Survey development

Two surveys were developed by the study authors: one for PDs and one for RGs. Survey content for both surveys was determined based on (1) the epidemiology of presenting complaints and disease states that present to US emergency departments, (2) established evidence of sex and gender differences in these disease states, (3) the ACGME EM listing of the core content for emergency medicine residency training [10], and (4) current standards for sex- and gender-based medicine curricula objectives from other fields [5–8]. In developing the survey, as per the National Institutes of Health (NIH), women’s health was defined as pertaining to diseases or conditions unique to, more prevalent in, or more serious in women, including diseases for which manifestations, risk factors, or interventions differ in women [11]. The survey instrument for RGs was tested for clarity and feasibility by a group of 10 residents from the authors’ residency program who commented on content and format; the PD survey was adapted based on PD feedback as well. See Additional files 1 and 2 for both the PD and RG surveys.

### Survey content

Both surveys included a description of the study and assurances of the confidentiality of responses followed by a question asking potential respondents for their consent to participate. Participants did not enter any identifying information. The survey for the PDs included questions regarding general residency program information, allocation of curriculum time for content on sex and gender issues in EM, barriers to SGBM curriculum development and implementation, assessment of EM curriculum priorities, types of learning activities that could be easily incorporated into residency curricula, and settings in which residents should learn the most about gender medicine. The survey sent to RGs assessed whether they felt instruction in SGBM was relevant to their training and whether their instruction in SGBM had been adequate. Preferred learning activities and settings for learning about gender medicine were also assessed. Demographic and practice information was requested including gender and predominant post-graduate practice setting. Both surveys were made available online by the study institution’s technical support group, and survey links were placed on a hospital-affiliated website. For both PDs and RGs, question responses were in the form of multiple choice questions and three-point Likert scales [12]. For all three-point Likert scales, response options were “yes,” “somewhat,” and “no.”

### Data analysis

Results of both PD and RG surveys were automatically and anonymously downloaded into an excel spreadsheet, and completion was tracked by survey number and not directly linked to the individual survey participants. Descriptive statistics were used to describe RGs’ responses about SGBM in their residency training, RGs’ responses regarding the relevance of SGBM in their current practice, RGs’ perspectives on preferred learning activities, and the settings in which RGs learned about gender medicine. Descriptive statistics were also used to report PDs’ responses about SGBM as a curriculum priority, PDs' responses about perceived barriers to implementing SGBM, PDs’ perspective on the most feasible types of learning activities for residents, and settings in which PDs felt residents should learn the most about gender medicine.

Specifically, RGs were asked if they had been trained to take gender into account in the presentation and management of specific conditions. The number of participants who responded with “yes,” “somewhat,” and “no” on the three-point Likert scale as above were tabulated. Next, RGs were asked if they had received adequate training on gender differences in emergency conditions. Those responding “yes” to this question (as opposed to “somewhat” or “no”) were considered to have received adequate training. Finally, RGs were asked if gender differences in emergency conditions were relevant to their practice. Participants who responded “yes” were compared to those who responded “no” and those who responded “somewhat.” PDs were asked whether their residency curricula included explicit training on how sex and gender influenced specific conditions. The number of participants who responded with “yes,” “somewhat,” and “no” on the three-point Likert scale as above were tabulated. PDs were also asked whether gender differences in disease presentation and management were a priority in the training curriculum for the clinical practice of emergency medicine. If PDs responded “yes” (as opposed to “somewhat” or “no”), they were included in the group that considered this a priority.

## Results

Of the 140 PDs, 54 of 140 (38.6 %) responded to the survey, while 226 of 2023 (11.2 %) RGs responded to the survey. Of the RGs, 42.7 % were female, most (70.5 %) had completed a 3-year residency program, and 54.3 % reported being in private practice (Table 1). Of the PDs, 74 % were PDs of 3-year EM residency programs (Table 1).

**Table 1:** Description of survey respondents

Residency graduates	% (*N*)
Female gender	42.7 % (94)
Type of residency training program	
1 through 4 programs	20.5 % (45)
2 through 4 programs	9.1 % (20)
1 through 3 programs	70.5 % (155)
Geographic area of residency training	
Northeast	45.0 % (98)
South	20.2 % (44)
Midwest	25.7 % (56)
West	9.2 % (20)
Current practice type	
Private	54.3 % (119)
Academic	24.7 % (54)
Combination	21.0 % (46)
Program directors	
Program type	
1 through 4 programs	20 % (10)
2 through 4 programs	6.0 % (3)
1 through 3 programs	74.0 % (37)
Geographic area of residency program	
Northeast	38.0 % (19)
South	22.0 % (11)
Midwest	28.0 % (14)
West	12.0 % (6)

Overall, less than half of the RGs answered “yes” when asked if they had received adequate training on sex and gender differences in emergency conditions (43.2 %, *n* = 95). Twenty-four participants (10.9 %) felt they had not received adequate training in this area (indicated by an answer of “no”), while 45.9 % (*n* = 101) responded with the answer “somewhat.” Six participants skipped the question.

When asked whether they had been trained to take gender into account in the presentation and management of specific conditions, the disease conditions with the lowest number of RGs answering “yes” were asthma/COPD (5.0 %, *n* = 11), carotid and vertebral artery dissections (10.5 %, *n* = 23), and pharmacokinetics (10.0 %, *n* = 22) (Table 2). The disease conditions with the highest number of RGs answering “yes” were acute coronary syndrome (80.1 %, *n* = 177), partner abuse (75.6 %, *n* = 167), and urinary tract disorders (71.5 %, *n* = 158). This was consistent with the results from the PDs’ surveys (Table 3). When program directors were asked whether their residency curriculum included explicit training about sex and gender differences in the presentation and management of specific disease states, most PDs reported that their programs had explicit training on partner abuse (82.0 %, *n* = 41), acute coronary syndrome (70.0 %, *n* = 35), and urinary tract disorders (68.0 %, *n* = 34) (as indicated by a response of “yes”). Few PDs reported having explicit training on pharmacokinetics (4 %, *n* = 2), asthma/COPD (6 %, *n* = 3), and vertebral/carotid artery dissections (10 %, *n* = 5).

**Table 2:** Resident graduates’ responses to survey item, “Have you been trained to take gender into account in the presentation and management of the following conditions?”

Disease condition	No (*n*, %)		Somewhat (*n*, %)		Yes (*n*, %)	
Endocrine disorders (diabetes mellitus, thyroid disease)	81	36.7 %	65	29.4 %	75	33.9 %
Acute coronary syndrome	3	1.4 %	41	18.6 %	177	80.1 %
Hypertension	119	54.1 %	60	27.3 %	41	18.6 %
Thromboembolic disease (DVT and PE)	65	29.4 %	53	24.0 %	103	46.6 %
Asthma/COPD	192	86.9 %	18	8.1 %	11	5.0 %
Trauma	76	34.5 %	61	27.7 %	83	37.7 %
Partner abuse	6	2.7 %	48	21.7 %	167	75.6 %
Carotid/vertebral artery dissections	162	73.6 %	35	15.9 %	23	10.5 %
Neurologic conditions (multiple sclerosis, myasthenia gravis, CVA, seizures, headache)	67	30.3 %	60	27.1 %	94	42.5 %
GI conditions (biliary disease, dyspepsia, IBS, appendicitis, abdominal pain)	55	24.9 %	64	29.0 %	102	46.2 %
Substance abuse	121	55.0 %	63	28.6 %	36	16.4 %
Depression/suicide	39	17.8 %	51	23.3 %	129	58.9 %
Urinary tract disorders	18	8.1 %	45	20.4 %	158	71.5 %
Sexually transmitted diseases	25	11.3 %	49	22.2 %	147	66.5 %
HIV/AIDS	144	65.5 %	46	20.9 %	30	13.6 %
Autoimmune disease	27	12.3 %	67	30.5 %	126	57.3 %
Pulmonary disease (sarcoidosis, PPH)	129	58.4 %	53	24.0 %	39	17.6 %
Osteoporosis/fracture management	41	18.6 %	44	19.9 %	136	61.5 %
Pain management	150	68.2 %	44	20.0 %	26	11.8 %
Pharmacokinetics	135	61.4 %	63	28.6 %	22	10.0 %
Communication styles	60	27.1 %	81	36.7 %	80	36.2 %

**Table 3:** Program directors’ responses to survey item, “Does your current residency curriculum include explicit training (didactic and clinical) about gender differences in the presentation and management of the following clinical or disease states?”

Disease condition	No (*n*, %)		Somewhat (*n*, %)		Yes (*n*, %)	
Endocrine disorders (diabetes mellitus, thyroid disease)	22	44.0 %	14	28.0 %	14	28.0 %
Acute coronary syndrome	3	6.0 %	12	24.0 %	35	70.0 %
Hypertension	30	62.5 %	11	22.9 %	7	14.6 %
Thromboembolic disease (DVT and PE)	21	42.0 %	10	20.0 %	19	38.0 %
Asthma/COPD	35	70.0 %	12	24.0 %	3	6.0 %
Trauma	12	24.0 %	13	26.0 %	25	50.0 %
Partner abuse	3	6.0 %	6	12.0 %	41	82.0 %
Carotid/vertebral artery dissections	38	76.0 %	7	14.0 %	5	10.0 %
Neurologic conditions (multiple sclerosis, myasthenia gravis, CVA, seizures, headache)	15	30.0 %	22	44.0 %	13	26.0 %
GI Disorders (biliary disease, dyspepsia, IBS, abdominal pain, appendicitis)	20	40.0 %	18	36.0 %	12	24.0 %
Substance abuse	29	58.0 %	13	26.0 %	8	16.0 %
Depression/suicide	8	16.0 %	14	28.0 %	28	56.0 %
Urinary tract disorders	3	6.0 %	13	26.0 %	34	68.0 %
Sexually transmitted diseases	5	10.0 %	12	24.0 %	33	66.0 %
HIV/AIDS	26	52.0 %	17	34.0 %	7	14.0 %
Autoimmune disease	17	34.0 %	20	40.0 %	13	26.0 %
Pulmonary disease (sarcoidosis, PPH)	33	66.0 %	11	22.0 %	6	12.0 %
Osteoporosis/fracture management	7	14.0 %	24	48.0 %	19	38.0 %
Pain management	32	64.0 %	10	20.0 %	8	16.0 %
Communication styles	21	42.0 %	14	28.0 %	15	30.0 %
Pharmacokinetics	36	72.0 %	12	24.0 %	2	4.0 %

**Fig. 1:**
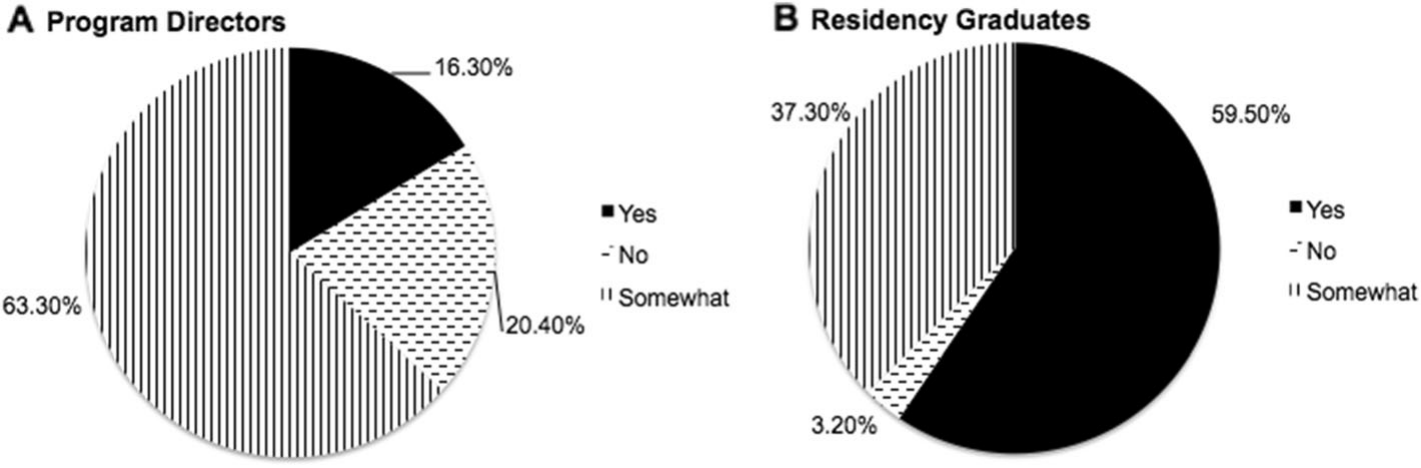
SGBM as a curriculum priority for program directors (PDs) vs. relevance of SGBM to EM practice as per residency graduates (RGs). *SGBM* sex- and gender-based medicine, *EM* emergency medicine

Figure 1 compares PDs’ responses to whether or not SGBM was a curriculum priority with the relevance of SGBM to EM practice as reported by RGs. In response to the question of whether gender differences in emergency conditions were relevant to their practice, 59.5 % (*n* = 131) of RGs answered “yes.” Conversely, only 16.3 % (*n* = 9) of PDs felt that gender differences in disease presentation and management were a priority in the training curriculum for the clinical practice of emergency medicine.

Table 4 describes the obstacles to incorporating SGBM into the curricula by PDs. The majority of PDs (76.6 %, *n* = 36) reported competing curricular demands as a major obstacle to incorporating SGBM into their residency’s curriculum. Other obstacles included lack of qualified faculty, faculty interest, evidence-based content, and clinical exposure (Table 2).

**Table 4:** Perceived obstacles to incorporating SGBM into EM residency curriculum

Potential Obstacle	% (n)
Competing curricular demands	76.6 % (36)
Lack of qualified faculty	21.3 % (10)
Lack of faculty interest	36.2 % (17)
Lack of resident interest	21.3 % (10)
Lack of evidence-based content	53.2 % (25)
Lack of clinical exposure	2.1 % (1)
Other (please specify)	8.5 % (4)

*SGBM* sex- and gender-based medicine, *EM* emergency medicine

When asked what type of learning activities they preferred, RGs’ responses were fairly equally distributed across choices. Most chose large group activities (*n* = 62, 28.2 %), but similar numbers chose small group activities (*n* = 55, 25 %), clinical activities (*n* = 47, 21.4 %), and self-study (*n* = 55, 25.0 %). In contrast, over half of PDs (*n* = 26, 53.1 %) felt that large group activities would be most easily integrated into residency training. Less PDs chose small group activities (*n* = 14, 28.6 %), clinical activities (*n* = 2, 4.1 %), and self-study activities (*n* = 5, 10.2 %).

**Fig. 2:**
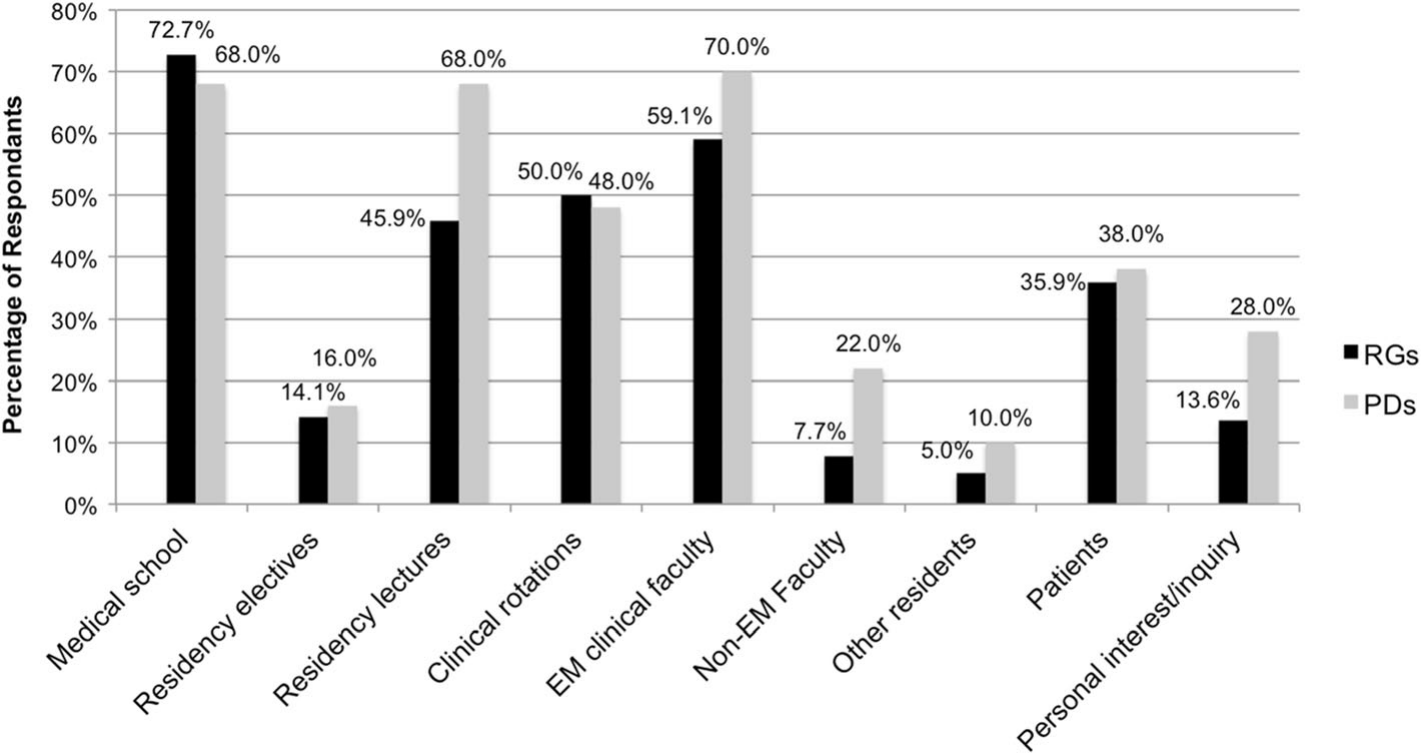
Settings in which resident graduates (RGs) learned the most SGBM vs. program directors’ (PDs) responses describing where residents should learn the most about SGBM

Finally, Fig. 2 shows RGs’ responses describing where they learned the most about women’s health issues and gender medicine and compares them to PDs’ responses describing where they felt residents *should* learn the most about gender medicine. Of note, 68.0 % of PDs felt residents should learn about gender medicine in residency lectures, yet only 45.9 % of RGs reported that they learned about these topics in residency lectures. In addition, there were discrepancies between the numbers of PDs who felt residents should learn about gender medicine from non-EM faculty (22.0 %) or through personal inquiry (28.0 %) and the number of RGs who reported that they learned about gender medicine from non-EM faculty (7.7 %) or personal inquiry (13.6 %).

## Discussion

In our educational needs assessment of whether EM RGs are receiving adequate training in SGBM, the majority of RGs felt they had not received adequate training on gender differences in emergency conditions. This suggests a need for sex- and gender-based curricula that may not be met by current EM residency training programs.

Content areas in which RGs felt that they had received the most instruction included acute coronary syndrome, partner abuse, and urinary tract disorders. Asthma/ COPD, vertebral and carotid artery dissections, and pharmacokinetics were the areas in which RGs felt that they had received the least amount of instruction overall. These responses are consistent with common SGBM topic areas within curriculum programs outside of the specialty of EM. For example, SGBM curriculum in internal medicine training programs is often limited to gender differences in cardiovascular disease [5]. Future curriculum development for EM residencies and fellowships should include content on all health and disease states in which research has shown sex and gender differences. For example, in regard to pharmacokinetics, one subject area in which RGs reported the need for more instruction, it is of critical importance for EM physicians to be aware of sex differences as this affects current dosing recommendations and medication responses. This has been seen with sex differences in the metabolism of zolpidem that led to a recommendation to decrease the dose for women only by 50 % [13].

In our study, PDs perceived many obstacles to incorporating sex- and gender-based content into residency curricula, with competing curricular demands being the most frequently cited obstacle. In order to ensure that sex- and gender- specific curriculum is taught to EM residents, steps should be taken to teach PDs simple and feasible ways to add this content to already existing curricula. For example, discussions about the potential influence of sex and gender on a variety of clinical questions can be easily and efficiently incorporated into both formal and informal residency didactics [9]. Another commonly cited obstacle, lack of faculty with expertise to teach SGBM, could be addressed by creating multidisciplinary programs and working with experts from other departments [7]. In future surveys of PDs, it may be helpful to have PDs rank the barriers to the incorporation of SGBM into residency curriculum in order to better design solutions to overcome these barriers. In addition, future research should be devoted to designing effective solutions for PDs to overcome such barriers as these obstacles have the potential to significantly delay universal adoption of a SGBM curriculum.

Our findings of RGs’ preferred types of learning activities compared to the types of learning activities that PDs felt would be most easily incorporated into residency curricula suggest some challenges to incorporating sex and gender content into residency curricula. Our results suggest that RGs have a wide range of preferences for learning activities, but over half of PDs felt large group activities such as residency conference would be the easiest type of learning activity to incorporate SGBM content. In order to adequately teach residents about SGBM, PDs should consider using a variety of learning activities to appeal to the different learning styles of as many residents as possible. Our findings regarding where RGs reported learning about gender medicine suggest that PDs may be overestimating the effectiveness of residency conferences to teach residents about SGBM. Our results may also suggest that some PDs think residents should learn SGMB from other, non-EM faculty or through personal inquiry, but most RGs indicated they are not learning about SGBM in these ways. Based on these findings, PDs may need to be educated as to the importance of including SGBM into EM residency curriculum.

As sex- and gender-specific research in EM progresses, we must continue to monitor and measure whether these principles are being incorporated into EM residency curricula. Such work is supported by experts from both our own specialty as well as the Institute of Medicine [14]. Since our survey study was conducted, our specialty has published recommendations for the incorporation of sex and gender into EM research and residency curricula [15, 16]. We believe this reflects an increasing level of awareness among emergency physicians of the influence of sex and gender on conditions commonly diagnosed and managed in the ED. Future studies should investigate potential changes over time in PDs’ and RGs’ attitudes towards the need for SGBM content in EM residency curricula and investigate the effectiveness of incorporation into different types of educational formats; for example, simulation may be a practical way of delivering SGBM content in a meaningful, innovative way.

Our study has several limitations. Because of the relatively low survey response rates, our results may be subject to response bias. Specifically, those PDs and RGs who chose to respond may have had higher levels of interest in SGBM than other providers; if so, our measurements of participants’ attitudes towards the relevance of SGBM to the practice of EM may not be representative of the overall population of emergency medicine providers. Our response rates may also limit the generalizability of our study results. In future studies of SGBM in residency curriculum, non-responders could be compared to responders or to national data with regard to demographics and other baseline characteristics in order to increase generalizability. Additionally, the survey was sent to ACGME accredited residency programs. Future surveys should also be sent to American Osteopathic Association (AOA) residency programs in order to adequately represent all EM residency programs. It is important to note that RGs may have experienced recall bias in their recollection of SGBM curriculum in their training. It is possible that RGs may have gained knowledge of sex and gender differences in specific disease conditions from sources other than residency curricula. Finally, though both survey instruments were developed using existing literature on SGBM as well as EM residency core content, the survey instruments have not been previously validated. Despite the inherent limitations of our study, the results have the potential to guide future studies regarding attitudes towards the inclusion of SGBM into EM residency curricula.

## Conclusions

In summary, most RGs of ACGME-accredited EM residency programs felt that their instruction in SGBM was not adequate, and SGBM was not reported as a consistent priority among PDs. In order to translate our knowledge of sex and gender differences in emergency conditions into improving patient care, SGBM must be incorporated into the curricula of our trainees.

## Additional files

Additional file 1: Program-Directors. (PDF 234 KB)
Additional file 2: Resident Graduates. (PDF 229 KB)
