# Supplementary material for: Training emergency physicians in sex- and gender-based medicine: assessing attitudes of program directors and residency graduates
Source: Biol Sex Differ. 2016 Oct 14;7(Suppl 1):48. doi: 10.1186/s13293-016-0098-2 (PMC5073786; doi:10.1186/s13293-016-0098-2)
Supplement: Additional file 1: — Program-Directors. (PDF 234 KB) [file 13293_2016_98_MOESM1_ESM.pdf]

Emergency Medicine Research

Survey: Are we training emergency physicians to practice gender medicine?

BrownMed
BROWN MEDICAL SCHOOL

Brown University National Center of Excellence in Women's Health

We would like to ask you to participate in a research study called "Are we training emergency physicians to practice gender medicine?" that will assess your training in recognizing and treating gender differences in emergency conditions. Your completion of the survey will take 3-5 minutes of your time. There are 10 questions we would like you to answer. This survey is the only thing that will be asked of you. There are no questions that should cause you any discomfort. Your taking part in this survey is completely voluntary. If you do not want to complete the survey you are free to choose not to fill it out.

The results of this survey will not benefit you personally. However, once you have completed the survey you will be entered into a drawing for a $100 Amazon.com gift certificate that will be awarded to 10 participants that will be selected in a random drawing. We are hoping the results of this survey will help us to better develop curricula in gender medicine for postgraduate emergency medicine training. The responses to the survey will be confidential. None of the information you will provide will be linked to any of your personal information.

If you have questions about the research study please contact any of the researchers listed below. If you have any questions about your rights as a research subject please feel free to call our office of research administration manager, Patricia Houser at 401-444-2099.

Thank you for your participation.

Please select one of the following surveys:

- **Survey for ACEP Young Physicians**
- **Survey for Program Directors and Staff**

Deborah Gutman, MD, MPH
Alyson McGregor, MD, MA
Elizabeth Sutton, MD
Elizabeth Nestor, MD

Back | More about Rhode Island Hospital

Exit this survey >>

Lifespan

Rhode Island Hospital

Advancing Medicine. *Touching Lives.*

Program Survey: Are We Training Emergency Physicians to Practice Gender Medicine?

* **1. Please enter your assigned survey number (included in your e-mail invitation):**

Next >>

Lifespan

Rhode Island Hospital

Advancing Medicine. *Touching Lives.*

Program Survey: Are We Training Emergency Physicians to Practice Gender Medicine?

2. Does your current residency curriculum include explicit training (didactic and clinical) about GENDER DIFFERENCES in the presentation and management of the following clinical or disease states:

	No	Somewhat	Yes
Acute coronary syndrome			
Asthma/COPD			
Autoimmune disease			
Carotid/Vertebral artery dissections			
Communication styles			
Depression/Suicide			
Endocrine disorders (Diabetes mellitus, Thyroid disease)			
GI Disorders (Biliary disease, Dyspepsia, IBS, Abdominal pain, Appendicitis)			
HIV/AIDS			
Hypertension			
Neurologic conditions (Multiple sclerosis, Myasthenia gravis, CVA, Seizures, Headache)			
Osteoporosis/fracture management			
Pain management			
Partner abuse			
Pharmacokinetics			
Pulmonary disease (sarcoidosis, PPH)			
Sexually transmitted diseases			
Substance abuse			
Thromboembolic disease (DVT and PE)			
Trauma			
Urinary tract disorders			

<< Prev   Next >>

Program Survey: Are We Training Emergency Physicians to Practice Gender Medicine?

3. Do you believe that gender differences in disease presentation and management are a priority in the training curriculum for the clinical practice of emergency medicine?

- No
- Somewhat
- Yes

4. Do you feel that you have faculty qualified to teach/train residents in gender medicine?

- No
- Somewhat
- Yes

5. Check any barriers you perceive to implementing a gender medicine curriculum into your training program:

- Competing curricular demands
- Lack of qualified faculty
- Lack of faculty interest
- Lack of resident interest
- Lack of evidence based content
- Lack of clinical exposure
- Other (please specify)

6. Which curricular format would be most easily integrated into your training program?

- Large group activities (e.g., grand rounds, conferences and workshops)
- Small group activities (e.g., journal clubs, mentored research, rotation-based small group sessions)
- Clinical activities (e.g., general clinic, specialty clinic and ward rotations)
- Self-study activities (e.g., online modules, textbooks, electives)
- Other (please specify)

7. Where and from whom do you feel residents should learn the most about gender differences in medicine? (Check all that apply.)

- Medical school
- Residency electives
- Residency lectures
- Clinical rotations
- EM clinical faculty
- Non-EM faculty
- Other residents
- Patients
- Personal interest/inquiry
- Other (please specify)

<< Prev   Next >>

Rhode Island Hospital

Advancing Medicine. *Touching Lives.*

Lifespan

Program Survey: Are We Training Emergency Physicians to Practice Gender Medicine?

8. Type of residency program:

- 1-3
- 2-4
- 1-4

9. Geographic area of residency training:

- Northeast (CT, MA, ME, NH, NJ, NY, PA, RI, VT)
- South (AL, AK, DE, DC, FL, GA, KY, LA, MD, MS, NC, OK, SC, TN, TX, VA, WV)
- Midwest (IL, IN, IO, KS, MI, MN, MO, NE, ND, OH, SD, WI)
- West (AK, AZ, CA, CO, HI, ID, MT, NM, OR, UT, WA, WY)

10. Additional comments:

<< Prev   Done >>

Emergency Medicine Research

Survey: Are we training emergency physicians to practice gender medicine?

Thank you for participating in our survey!

Thank you for taking the time to complete this survey. The results will be used to improve graduate medical education in emergency medicine.

Winners of the **$100 Amazon.com gift certificates** will be notified by mail (ACEP Young Physicians) or by email (residency program directors).

Back | More about Rhode Island Hospital
